# Large-scale intrinsic connectivity is consistent across varying task demands

**DOI:** 10.1371/journal.pone.0213861

**Published:** 2019-04-10

**Authors:** Paulina Kieliba, Sasidhar Madugula, Nicola Filippini, Eugene P. Duff, Tamar R. Makin

**Affiliations:** 1 Institute of Cognitive Neuroscience, University College London, London, United Kingdom; 2 FMRIB Centre, University of Oxford, Oxford, United Kingdom; 3 Oxford Centre for Human Brain Activity, Wellcome Centre for Integrative Neuroimaging, Department of Psychiatry, University of Oxford, Oxford, United Kingdom; University at Buffalo, UNITED STATES

## Abstract

Measuring whole-brain functional connectivity patterns based on task-free (‘resting-state’) spontaneous fluctuations in the functional MRI (fMRI) signal is a standard approach to probing habitual brain states, independent of task-specific context. This view is supported by spatial correspondence between task- and rest-derived connectivity networks. Yet, it remains unclear whether intrinsic connectivity observed in a resting-state acquisition is persistent during task. Here, we sought to determine how changes in ongoing brain activation, elicited by task performance, impact the integrity of whole-brain functional connectivity patterns (commonly termed ‘resting state networks’). We employed a ‘steady-states’ paradigm, in which participants continuously executed a specific task (without baseline periods). Participants underwent separate task-based (visual, motor and visuomotor) or task-free (resting) steady-state scans, each performed over a 5-minute period. This unique design allowed us to apply a set of traditional resting-state analyses to various task-states. In addition, a classical fMRI block-design was employed to identify individualized brain activation patterns for each task, allowing us to characterize how differing activation patterns across the steady-states impact whole-brain intrinsic connectivity patterns. By examining correlations across segregated brain regions (nodes) and the whole brain (using independent component analysis) using standard resting-state functional connectivity (FC) analysis, we show that the whole-brain network architecture characteristic of the resting-state is comparable across different steady-task states, despite striking inter-task changes in brain activation (signal amplitude). Changes in functional connectivity were detected locally, within the active networks. But to identify these local changes, the contributions of different FC networks to the global intrinsic connectivity pattern had to be isolated. Together, we show that intrinsic connectivity underlying the canonical resting-state networks is relatively stable even when participants are engaged in different tasks and is not limited to the resting-state.

## Introduction

Functional connectivity (FC) is a powerful and widely used tool for probing brain network organization and function in healthy [1–5] and clinical populations [6–11]. Many studies focus on FC measured during rest, which can be accurately described by a relatively small number of spatiotemporal patterns that remain consistent across different participants and datasets [12]. The spatial composition of these resting-state patterns, often referred to as intrinsic connectivity networks (ICNs) or resting state networks, have been shown to mirror the respective brain states during task execution [9, 13–16].

The high correspondence between rest- and task-based FC patterns [4, 14, 17–19], as well as the changes in FC patterns between neurotypical and abnormal individuals, have led researchers to suggest that resting-state FC reflects the underlying synaptic efficacies in cortical networks [20–24]. That is, intrinsic FC is suggested to reflect the habitual state of the brain, independent of the specific context. However, many new studies, such as those employing psychophysiological interactions (PPI) [25], emphasize the differences in FC patterns resulting from dynamic changes in task demands [26–30]. These divergent observations raise the question of whether FC, as measured using fMRI, is sensitive to changes in brain activation. In other words, does intrinsic FC reflect the canonical (default, activation-independent), or current (transient, activation-dependent) state of the brain?

In this study, we sought to shed some light on this matter by testing the consistency of the ICN patterns across various well-defined steady-state tasks. We hypothesized that if intrinsic FC represents the canonical state of functional brain organization (i.e. synaptic efficacy) [21], it should remain relatively stable across changing tasks. Alternatively, if fMRI FC represented the transient task-dependent organization of the brain [26, 27], intrinsic connectivity would be expected to change, depending on activation changes within the networks. We focus our analysis on four steady-state conditions, collected either during rest or during three continuous tasks (without rest periods), allowing us to make inferences about resting and task-derived FC patterns based on the entire scan, rather than on brief rest and task periods used in traditional block designs. This unique design also allowed us to employ multiple fMRI analyses developed for studying resting-state FC (otherwise not suitable for the more standard block-design), to study task-state FC and minimized the influence of rest on the task-based ICNs. Note that steady-state scans have been previously shown to be less susceptible to confounding factors than block designs [31] and to produce more consistent FC results [3].

Previous studies have highlighted high degree of spatial overlap between rest and task-derived FC networks [14]. Here we took a step further and interrogated connectivity strength between distinct brain regions to determine whether intrinsic connectivity remains stable across task and rest states. The different active steady-states used in our study were chosen based on a factorial design (motor/visual on/off) and were designed to target well-characterized and robust activation profiles in distinct sets of brain areas, with high consistency within and across participants. The natural vision condition was designed to activate the entirety of the Occipital- and Lateral Visual canonical ICNs. The motor task was performed with the right hand and was thus designed to only activate the left sensorimotor cortex, which comprised a portion of the bilateral sensorimotor ICN. The visuomotor condition was designed to simultaneously activate both visual and motor nodes, affiliated with distinct canonical ICNs. A fifth task, designed to increase attentional task demand was also included, as detailed in the S1 Supporting Information. In addition to the 5-minute steady-state scans, we also used a traditional task-activation localizer (30 second blocks interleaved with baseline periods, see further details in the Methods) to measure changes in mean brain activation (BOLD signal level) induced by each of the tasks employed in the steady-state scans, and in each participant, allowing for participant-specific, customized analysis.

We first used these data to determine the relationship between steady-state task-induced activation (based on the localizer task) and the FC profile (based on the various steady state scans) across brain nodes. We then employed a data driven approach based on independent components analysis (ICA) to investigate the stability of the ICNs’ spatial profile, given the changed input induced by each steady-state task. For this purpose, we utilized the resting-state dataset collected by the Human Connectome Project as a model of the resting state ICNs [32]. Both of these analyses showed little modulation in whole-brain FC patterns based on changed task activation. Finally, we used dual-regression analysis [7] to isolate network-specific local changes in connectivity, induced by different task-demands and activation profiles. By demonstrating that the overall architecture of the ICNs is highly robust despite changing task demands and specific localized changes in FC, we conclude that intrinsic FC largely reflects the a priori habitual state of the brain, independent of the specific context.

## Methods

### Participants and experimental design

15 healthy volunteers (7 females, 8 males, age = 27.25±4.4yr, all right handed) without any previous neurological disorders participated in this study. The study was approved by the NHS national research ethics service (10/H0707/29). All participants gave their written informed consent before participating in the study. All participants underwent steady-state fMRI along with task localizer fMRI, with the order of the scans randomly determined. Due to an error in the block design acquisition, one participant was discarded from all analyses involving the task localizer data (i.e. the node analysis). We note that the group ICNs in our dataset were comparable to those obtain using large datasets (HCP, see below), indicating adequate statistical power for the FC analysis. This dataset has been previously used to investigate related research questions [33–35].

**Fig 1:**
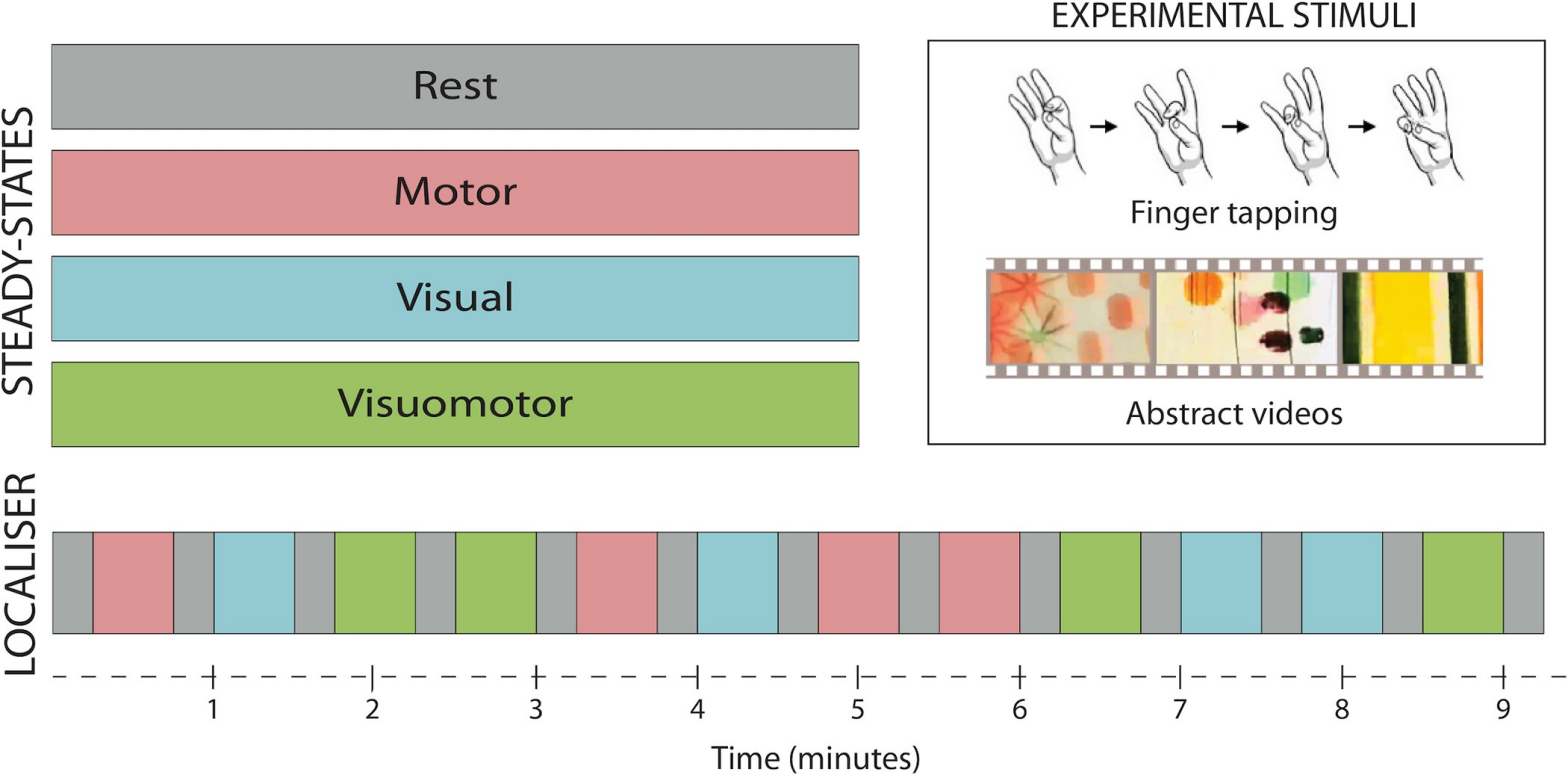
Steady-states design. Participants underwent a set of separate task-based (visual, motor and visuomotor) or task-free (resting) steady-state scans, each acquisition lasting five minutes. In addition, a task-localizer scan was employed to identify brain activations induced by each of the steady-state tasks.

Participants were scanned under four separate, five-minute continuous steady-state conditions (with no baseline epochs): rest, motor only, visual only, and simultaneous (but independent) visual and motor tasks (Fig 1). The motor condition involved continuous sequential finger tapping against the thumb, using the right hand. Participants were asked to maintain a tapping frequency of 1Hz, and tapping pace was practiced prior to the scan. The natural vision condition consisted of videos of colorful abstract shapes in motion (S1 Video), modified from the work of the artist Len Lyn (circa 1930’s). During the combined visuomotor condition participants viewed the aforementioned videos while simultaneously performing the self-paced motor tapping task. The usage of ecologically-valid “low-level” tasks allowed us to construct a factorial design for activation profiles (i.e. orthogonal/additive activation in visual and motor areas across steady-states). In an additional fifth scan, described in the S1 Supporting Information section, participants repeated the combined visuomotor condition, but were asked to change the finger-tapping direction (index-to-pinkie and reverse) whenever they noticed a monochrome frame inserted in the video. This task was designed to explore the role of attentional load on the ICNs’ integrity. The order of scans was counterbalanced across participants, such that different participants were presented with the various scans using different, but complementary, order. A fixation cross was presented in all conditions and participants were asked to keep their eyes on the cross throughout the study.

An additional task-activation localizer scan was performed under the same main task conditions to identify participant-specific changes in average activation levels during each of the tasks. This scan used pseudo-randomized block design consisting of 30 second task intervals separated by 15 second baseline periods. During the localizer scan, each of the three main study tasks (visual only, motor only, visuomotor) was repeated four times for a total scan time of 9 minutes and 15 seconds (Fig 1).

### Data acquisition

Functional data were acquired in a Siemens Vario 3T scanner, using a 32-channel head coil and a high-resolution multiband (factor 6) sequence with the following parameters: voxel size = 2mm isotropic, TR = 1300ms, TE = 40ms, flip angle = 66° [36, 37]. 72 slices with 2mm thickness and no slice gap were acquired in the oblique axial plane, covering the whole cortex and cerebellum. Total number of volumes acquired: 230. For the task-localizer, the blood-oxygenation level dependent (BOLD) fMRI signal was acquired using a multiple gradient echo-planar T2*-weighted pulse sequence, with the parameters: voxel size = 3mm isotropic, TR = 3000ms; TE = 30ms; flip angle = 90°; imaging matrix = 64×64; FOV = 192mm axial slices. Forty-six slices with slice thickness of 3mm and no gap were acquired in the oblique axial plane, covering the whole cortex, with partial coverage of the cerebellum. Total number of volumes acquired: 185. Anatomical data were acquired using a T1-weighted magnetization prepared rapid acquisition gradient echo sequence (MPRAGE) with parameters: TR: 2040ms; TE: 4.7ms; flip angle 8°; 1mm isotropic resolution. Field maps were obtained in order to reduce spatial distortion of the EPI images.

### Data pre-processing

All imaging data were processed using FSL-FEAT (version 6.00) [38]. Pre-processing included motion correction, field-map correction [39] and brain extraction [40]. Localizer scans only were subjected to spatial smoothing using a Gaussian kernel of FWHM of 5mm. Following the Human Connectome Project’s (HCP, http://www.humanconnectomeproject.org) minimal processing protocol, no spatial smoothing was applied to the steady-states data [41]. To account for the influence of any non-neuronal contribution to the BOLD signal, steady-state data were additionally cleaned using FIX (FMRIB's ICA-based Xnoiseifier) [42] automated denoising. FIX was trained on a data from an identical multiband resting-state protocol, acquired on the same scanner [42, 43]. EPI volumes were spatially realigned to the mean image and co-registered with the structural T1-weighted image using Boundary-Based Registration [44]. Time-course pre-whitening was carried out using FILM (FMRIB's Improved Linear Model) with local autocorrelation correction [38]. All structural and functional images were registered to standard MNI using both linear (FLIRT) and non-linear (FNIRT) registration. Images underwent mean-based intensity normalization and high-pass temporal filtering (0.01Hz for steady-state scans; 0.005Hz for localizer scans).

### Task localizer

**Fig 2:**
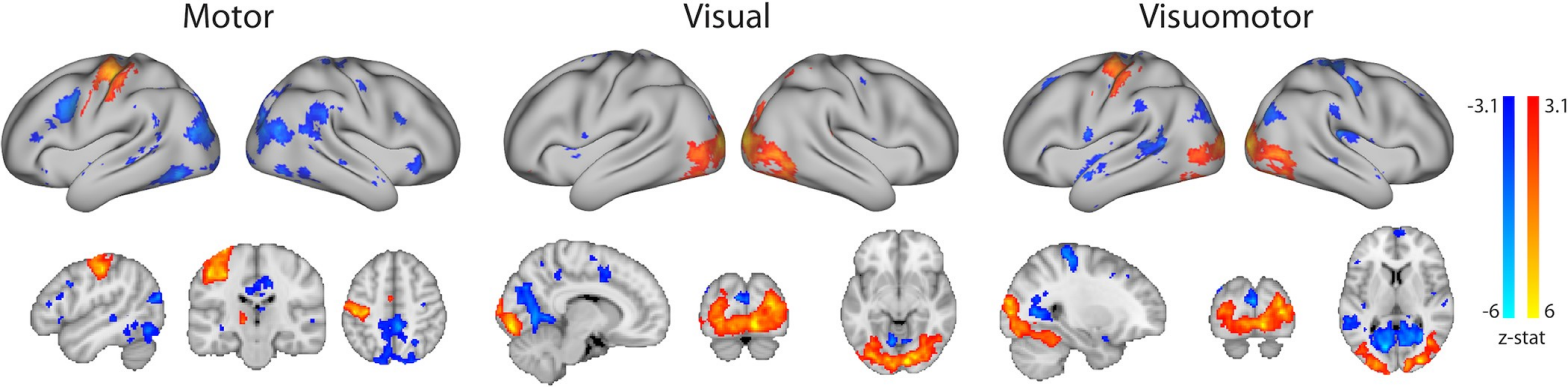
Group activation maps corresponding to each of the steady-states employed in the task localizer scan. All activation foci are projected onto the inflated surface of a template brain as well as on three anatomical planes.

A multi-level general linear model (GLM) analysis of the pre-processed localizer scans was used to identify regions that were activated during one or more of the conditions, relative to rest [45]. The block design time-course of each of the four steady-state conditions was convolved with the gamma function, and together with its temporal derivative used to model the activation time-course in individual participants. Two participant-level contrasts were defined for each of the task conditions (motor, visual, visuomotor) vs rest (task>rest and rest>task), resulting in a total of 6 contrasts (2 per each task condition). A high-level group analysis was performed using a mixed effects model (Fig 2). Z (Gaussianised T/F) statistic images were thresholded using clusters determined by Z>3.1 and a family-wise-error corrected cluster significance threshold of p<0.05 was applied to the suprathreshold clusters.

To verify that the regions activated during the task localizer were also activated during the steady-state scans we carried out a region of interest (ROI) analysis. The group task-localizer maps from motor, visual and visuomotor (task>rest) task conditions were thresholded (Z>3.1), binarized and used as the ROIs. The steady-states time-courses of the rest, motor, visual and visuomotor conditions were extracted from under the corresponding task-based ROIs separately for each participant/condition. The average amplitude of the entire time-course was compared between the rest and each of the tasks steady states using paired t-tests. Note that in this analysis a different pre-processing procedure was applied to the steady-states scans, including head-motion correction, field map correction, brain extraction, high-pass filtering, and spatial smoothing with 5 FWHM kernel. This change in pre-processing was motivated by the fact that this particular analysis was aimed at validating the activity changes, rather than connectivity changes within the steady-states data.

### Node parcellation and network matrix generation

This analysis aimed to determine whether changes in the intrinsic connectivity between task-related and task-unrelated nodes are associated with task-induced changes in mean fMRI activation across nodes. In short, we calculated a pairwise correlation between each nodes' time-courses in each of the conditions (rest, motor, visual, visuomotor). For each task-condition, pairs of nodes were then sorted based on whether both nodes, one node or neither of the nodes in each pair were positively activated during the visual, motor or visuomotor task localizer.

In detail, to measure inter-regional FC changes across the entire brain in each steady-state condition, an automatic multi-modal surface-based brain parcellation provided by the HCP [46] and comprising 180 cortical regions in each hemisphere was used. Upon initial inspection, the HCP parcellations were too coarse to successfully dissect the individual brains into task-related and task-unrelated nodes. This was because the task-activation clusters were often smaller in size or not perfectly aligned to the individual HCP ROIs (Fig B in S1 Supporting Information). Therefore, in order to generate participant- and condition-specific parcelattions which would allow us to correctly classify each parcellation as task-relevant or task-irrelevant, we needed to further customize the HCP ROIs. The HCP parcellations were dissected based on individual participants’ thresholded (Z>2.3) and binarized localizer scans (task>baseline). In other words, if the HCP ROI was found to be partially overlapping with a task activation cluster, that ROI was split into two separate nodes, each containing only activated/not activated voxels. This allowed us to generate nodes that are either task-relevant or task-irrelevant for each participant and condition (motor, visual, visuomotor). Note that as a result, the number of generated nodes (ROIs) was different for each individual and condition depending on their specific task activation pattern. Functional connectivity matrices were then created by correlating the average time-course of each ROI with the average time-course of each of the other ROIs, separately for each individual participant and for each condition. Defining the nodes individually for each participant (based on their activation maps in conjugation with HCP parcellation) allowed us to take into account the inter-participant functional variability. Note that the activation maps used for node definition (task-localizer) were not used in the node analysis, therefore avoiding circularity.

Next, we investigated whether changes in correlations between nodes during rest and each of the steady-state tasks were associated with changes in task-related activation. Specifically, we tested whether the changes in FC between a pair of nodes were associated with one or both of those nodes being activated [47]. First, to classify nodes based on their level of task-activation the following criteria were applied: A single node was classified as task-relevant (activated) if, based on a given individual participant’s task-localizer (task>baseline), the average z-statistic value (across all voxels within that node) was above 2.3. A node was labelled as task irrelevant (not activated) if, based on the same criterion, the mean z-statistic value was higher than -1 and lower than 1. Nodes producing values outside these criteria (including the deactivated nodes) were discarded from this analysis [47]. Note that the deactivated nodes were not analyzed as, to date, the physiological interpretations of negative BOLD modulations remain controversial [48–50]. Below we report results involving all of the study participants, but the same pattern of results was observed when excluding participants with low number of activated nodes in a given condition (if their number of activated nodes was lower than 1.5 interquartile range (IQR) below the lower quartile (Q1) of number of activated nodes across all participants).

**Fig 3:**
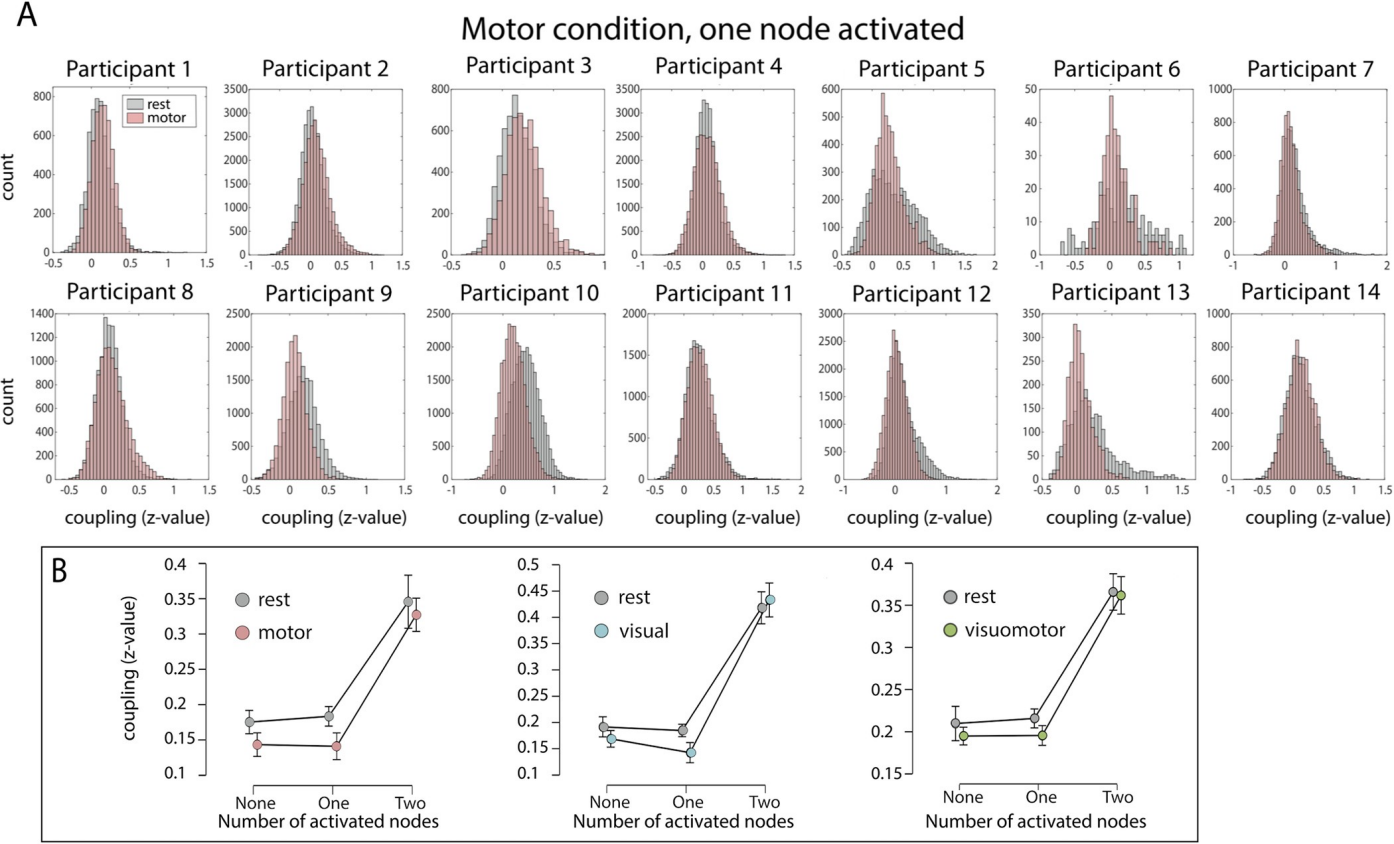
Relationship between task-evoked activation and differences between task-based and resting state FC. (A) Fisher z-transformed correlation coefficients between pairs of nodes, where only one of the nodes is activated during the motor steady-state condition, are displayed separately for each participant in the form of a histogram (y-axis depicting the number of node pairs in each bin; please note that the range of the y axis differs across individuals due to differences in the total amount of nodes activated during the task). If intrinsic connectivity changes with task activation, then FC should consistently decrease in the task-state, when only one of the nodes is activated, as compared to the resting-state. Although this is true for some participants (e.g. Participants 9 and 10), others show an opposite trend (connectivity increased in the motor condition, see e.g. Participants 1–3). (B) Relationship between number of activated nodes and mean FC for the three task comparisons (motor–pink, visual–blue, visuomotor—green). Note that while a significant main effect of the factor "number of activated nodes" (x-axis) can be observed, no significant interaction between activated nodes and task was found. Error bars indicate standard error of the mean.

Next, the correlation coefficients across each of the steady-state time-courses were calculated across all pair-combinations of the nodes. Each pair of nodes was then sorted based on whether both nodes, one node, or neither of the nodes were activated during the visual, motor or visuomotor conditions. The correlation values across those node-pairs were Fisher’s z-transformed and displayed in a histogram, separately for each participant, task (visual, motor and visuomotor) and number of activated nodes (no nodes activated/one node activated/two nodes activated, see Fig 3A and Figs C-J in S1 Supporting Information). Within each pair-category (none/one/two nodes activated) the average correlation coefficient (Fisher’s z-transformed) was further calculated for each participant and condition, and the mean values were analyzed using repeated-measures ANOVA and Bayesian repeated-measures ANOVA with number of task-related nodes in the pairwise correlation (none/one/two) and steady-state task (task/rest) as within-subject factors. This analysis was carried out in JASP [51], separately for motor, visual and visuomotor conditions. We note that averaging the FC across nodes may potentially mask more subtle connectivity changes within each node category, which was the focus of an additional specialized analysis (dual regression approach, see below).

### Correlations across intrinsic connectivity networks

In our next analysis, we investigated whether the spatial patterns of the resting-state connectivity networks correspond with those found during task states. For this purpose, we decomposed each of the steady-state datasets to identify 9 canonical networks of interests, and determined the extent of spatial correspondence across networks between the resting-state HCP dataset and each of our steady-states.

In detail, individual steady-state scans were temporally concatenated for each task condition (rest, motor, visual, visuomotor) to create task-specific 4D group datasets. Each of the datasets was decomposed to 50 independent components using MELODIC (Multivariate Exploratory Linear Optimized Decomposition into Independent Components, part of FSL software). Resulting group-level MELODIC output was matched, using spatial cross-correlations, to 10 canonical networks [14] obtained from the resting-state HCP data. The components with the highest correlation values from each task-specific MELODIC output were selected in a winner-takes all paradigm, to obtain 10 networks of interest independently for each of the 4 steady-state conditions.

**Fig 4:**
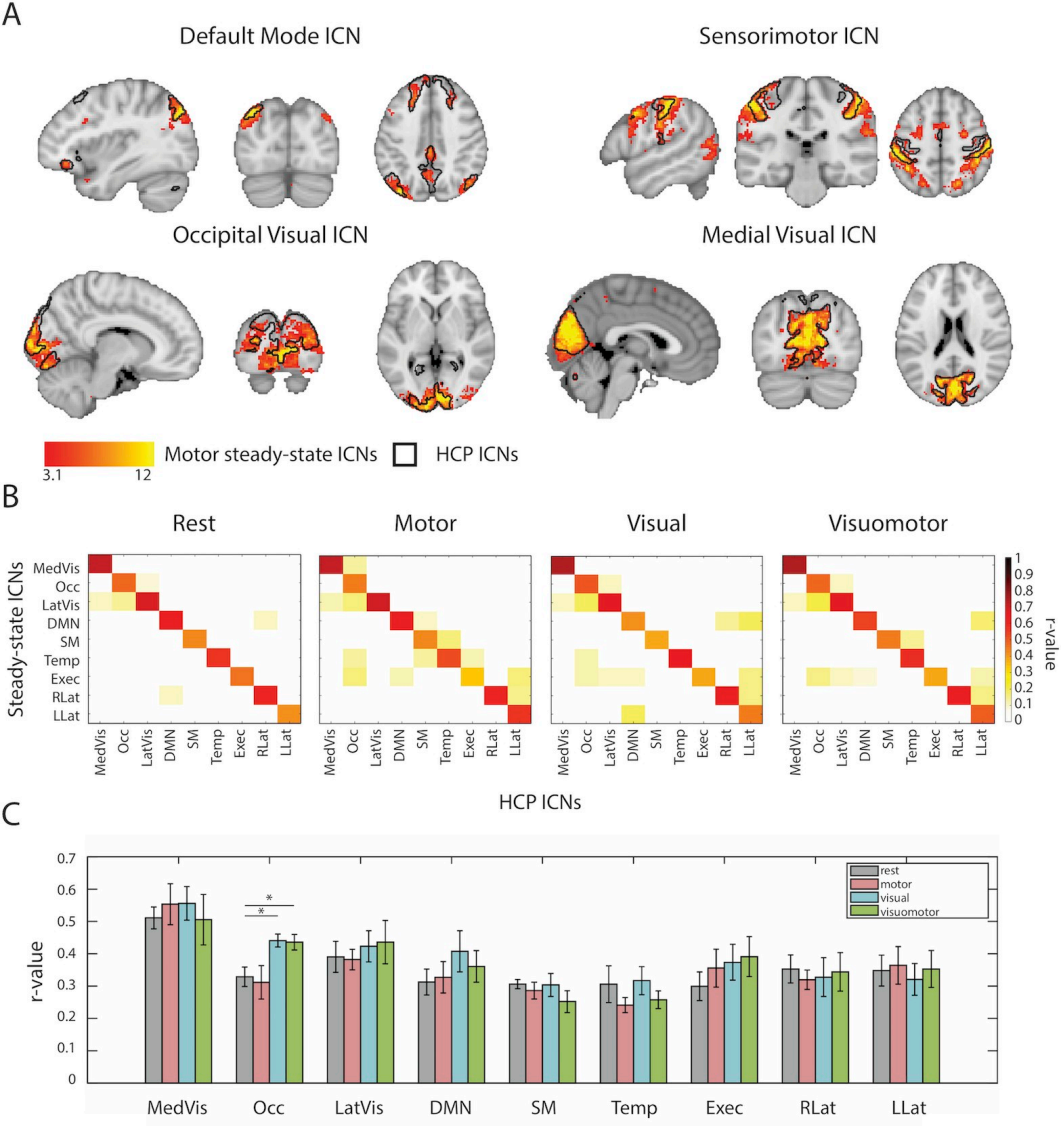
Spatial variability of ICNs between different steady-states. (A) Spatial maps of four task-related ICNs extracted from the motor condition (depicted in red-yellow scale) overlaid on the same ICNs extracted from the HCP data (depicted as black contours). (B) Whole brain correlation matrices of 9 major ICNs from the HCP data and their counterparts found in the steady-states data. Each ICN is correlated with all other ICNs. (C) Bar graph depicting mean spatial correlation coefficients (calculated from 100 bootstrapped ICA decomposition) of 9 major ICNs extracted from each of the steady-states conditions to their HCP counterparts. Note that only the Occipital Visual ICN (Occ) shows significant differences in its spatial correspondence to the HCP’s Occipital Network. Asterisks denote significance as determined using bootstrap percentile confidence intervals (see Methods). MedVis stands for Medial Visual ICN, Occ–Occipital Visual ICN, LatVis–Lateral Visual ICN, DMN–Default Mode Network, SM–Sensorimotor ICN, Temp–Temporal ICN, Exec–Executive ICN, RLat–Right Lateral ICN, LLat–Left Lateral ICN.

Upon inspection, the steady-state networks corresponding to the HCP cerebellum network were spatially diffuse in all four conditions, with its mean spatial correlation strength to the canonical cerebellum network being always under 0.2. The cerebellum network was therefore discarded from further analysis. Within each condition, the remaining 9 components were correlated against the HCP-derived networks across the entire brain (Fig 4B).

Next, for each steady-state condition, we investigated the spatial consistency of the 9 identified networks with the HCP resting-state networks. To this extent, the individual participants’ datasets were combined into surrogate group fMRI datasets using a bootstrapping procedure (i.e. random sampling with replacement). For each steady-state condition 100 surrogate datasets were created [12]. Each surrogate dataset contained data from ten randomly selected participants, drawn from the given steady-state. For each surrogate dataset, 50 independent components were extracted using MELODIC and based on the spatial cross-correlations matched to the canonical networks [14] obtained from the HCP’s data, resulting in 100 maps per condition for each of the 9 networks of interest. The obtained maps were spatially cross-correlated with the HCP-derived networks and for each network and each condition a bootstrapped sampling distribution of the r-values was built (Fig 4C).

To quantify the differences in spatial correspondence of the steady-state ICNs to the resting-state HCP-derived networks, we built bootstrapped distributions of difference between r-values corresponding to the rest and motor, rest and visual, and rest and visuomotor steady-states. For each of those difference distributions, r-values were normalized using Fisher’s z-transform and bootstrap percentile confidence intervals were calculated. Confidence intervals not overlapping with zero indicated significant difference between networks derived from rest and a given task condition in their spatial correspondence to the canonical ICNs.

### Dual regression analysis

Finally, we focused the analysis on more subtle changes in local connectivity strength, that might not have been detected using the previous analyses. Here we aimed at identifying voxel-wise connectivity changes associated with the four networks that overlapped with task-related activation changes during at least one of the employed steady-state conditions, as observed in the task-localizer: Default Mode Network ICN, Sensorimotor ICN, Occipital Visual ICN and Medial Visual ICN, as derived from the HCP dataset. We used the dual regression approach [7], allowing us to examine differences in voxel-wise connectivity strength within the networks of interest in each steady-state task condition. Importantly, unlike commonly used seed-based FC analyses, this approach allowed us to characterize the unique spatial distribution of each of the networks while accounting for variability shared with other networks.

In details, the main regressor of interest was the averaged time-course underlying the resting-state HCP component of interest, derived from each individual participant’s steady-state time-course, with individual voxels weighted based on their contribution to the group IC. The weighted ICA time-courses of the remaining HCP components of interest were also calculated within each individual participant, using the same procedure, and included as regressors of no interest, in a voxel-wise first-level GLM. The output values of this analysis are voxel-wise beta values, for each individual participant and in each steady-state, representing the strength of connectivity with the HCP components of interest, after accounting for partial contribution of all other ICs.

For group statistical analysis, these beta maps were compared between the steady-state conditions using a 2 (motor task on/off) by 2 (visual task on/off) ANOVA design. This analysis was carried out using FSL randomize non-parametric permutation testing, with 5000 permutations, using a threshold-free cluster enhancement approach [52]. This analysis was repeated independently for each of the four components of interest, to identify the strength of connectivity between individual voxels and the given network. Since none of the interactions were significant, the analysis was restricted to two main effects per network of interest, resulting in 8 comparisons. To account for multiple comparisons, we adjusted the alpha value to 0.00625 (Bonferroni correction).

## Results

### Task localizer

To evaluate changes in mean BOLD signal amplitude induced by each of the steady-state conditions (activation), we first examined group contrast maps for each of the task conditions versus rest, derived from the block-design localizer scan. As shown in Fig 2, the visual and motor conditions resulted in the canonical visual/motor fMRI activation patterns [53]. Since the finger-tapping motor task was performed using the right hand, during the motor condition positive activation could be observed in the left primary motor and pre-motor cortices (overlapping the Sensorimotor ICN), right cerebellum and the posterior part of the left putamen. As such, the activation profile only engaged a part of the canonical Sensorimotor ICN. In this condition, visual areas in the occipital cortex were associated with negative BOLD modulation (hereafter deactivation). The motor condition also resulted in deactivation, partially overlapping with areas formally known as comprising posterior regions of the Default Mode Network (e.g. posterior cingulate cortex, precuneus). The natural visual condition, involving abstract videos, activated low-level (foveal) and mid-level visual areas in the occipital and occipitotemporal cortex (overlapping with the Occipital Visual ICN) as well as higher-level visual areas in the occipitotemporal cortex (e.g. V4, hMT). This visual condition also resulted in deactivation in the low-level (peripheral) medial visual area (overlapping with the Medial Visual ICN) and in supplementary motor area. The combined visuomotor condition, involving both abstract videos and right-hand finger tapping, activated similar motor and visual areas as described for each condition separately, thereby providing an opportunity to study the combined activation impact across the individual visual and motor conditions. A comparison of the average time-course in the task-based and resting steady-state scans confirmed that the areas activated during the localizer were also activated during the steady state (motor vs. rest: t_(13)_ = 6.659; p<0.0001; visual vs. rest: t_(13)_ = 3.906; p = 0.0018; visuomotor vs. rest: t_(13)_ = 2.159; p = 0.05). The localizer data will be made available online at the Open Science Framework upon publication of the manuscript.

### Functional connectivity between nodes is not directly associated with changes in BOLD activation

To examine the large-scale relationship between activation and connectivity, we parcellated the brain of each individual participant into functional nodes (see Methods). If intrinsic FC is modulated by task demands (dependent on changes in node activation levels), then it should be increased/decreased in the task-state, when both/one of the nodes are activated by the task, respectively. This should result in a significant interaction between the number of nodes activated (zero, one, two) and the steady-state condition (task, rest). However, it is important to emphasize that this analysis is not suitable for identifying connectivity changes in specific node pairs, as the analysis is carried over the population of node pairs of the same affiliation.

We found that substantial proportion of FC changes is not significantly associated with activation. This was exemplified by a nonsignificant interaction between the number of activated nodes and the task (motor vs rest: F_(1.076,13.987)_ = 0.258, p = 0.637; visual vs rest: F_(1.266,16.458)_ = 1.666, p = 0.219; visuomotor vs rest: F_(1.083,14.074)_ = 0.357, p = 0.576). The Bayes Factor (BF) for the interaction was below 0.33 for all three conditions (motor vs rest BF: 0.285, visual vs rest BF: 0.282, visuomotor vs rest BF: 0.182; all versus rest), providing positive evidence in favor of the null hypothesis (no interaction) [54, 55].

We also found a significant main effect of the number of activated nodes in a pair (zero, one or two) on FC (Fig 3B), indicating that nodes that usually activate together tend to show increased connectivity independent of the steady state (motor vs rest: F_(1.268,16.485)_ = 44.643, p<0.001; visual vs rest: F_(1.07,13.908)_ = 50.267, p<0.001; visuomotor vs rest: F_(1.131,14.703)_ = 46.374, p<0.001). Finally, we found no significant main effect of task, showing that connectivity was not significantly different between task- and rest-states (motor vs rest: F_(1,13)_ = 1.327, p = 0.27; visual vs rest: F_(1,13)_ = 1.1688, p = 0.299; visuomotor vs rest: F_(1,13)_ = 0.477, p = 0.502). These null results were further examined using Bayesian statistics, where a threshold of Bayes factor (BF) < 1/3 was taken as positive (substantial) evidence in favor of the null hypothesis (no differences across networks) [55]. We found that the null hypothesis was supported for the visual (BF = 0.272) and visuomotor conditions (BF = 0.193), whereas the differences between the motor and rest conditions were ambiguous (BF = 0.46).

### Spatial consistency of ICNs across steady-state tasks

In the node analysis described above, we examined the local relationship between task-induced activation and FC. Although we found that the average FC between nodes was not reliably dependent on the activation changes between those nodes, it is possible that subtler changes in connectivity within each nodes category, impacting the overall spatial distribution of the intrinsic networks, were left undetected. In our next analysis, we thus aimed to examine the stability of the ICNs across different steady-state conditions. If ICNs spatial composition is strongly modulated by activation profiles, we would expect to see degraded correspondence between the task-based and classical resting-state ICNs.

All of the canonical ICNs were found in the ICA decomposition of each of the steady-state datasets. Moreover, spatial maps of both rest- and task-state networks showed high levels of consistency with the HCP-derived resting-state ICNs (see Fig 4A for task-relevant networks resolved from motor steady-state; see Figs K-O in S1 Supporting Information for all of the networks and conditions). Spatial correlations between congruent networks of the HCP and steady-state tasks were found to be considerably stronger than the correlations between incongruent networks (Fig 4B) (average r-value for the intra-network correlations: 0.54–0.55, average r-value for the inter-network correlations: 0.01–0.02), demonstrating that the overall spatial distribution of the networks is largely preserved across task-states. Note, however, that due to the whole-brain nature of this analysis, it is not suitable to identify highly localized changes (which we explore in our final analysis, see below).

To quantify the spatial overlap of the data-driven ICNs across steady-states with their HCP-counterpart, we ran a bootstrapping analysis (see Methods, Fig 4C), allowing us to quantify confidence intervals of spatial correlation values. In addition, this analysis provides an important opportunity to validate the quality of our data, considering our sample size was much smaller than that of the HCP. Despite the fact that HCP-derived ICNs were acquired during resting state while the task-based steady-states were acquired during activation of a range of brain areas (see Task localizer results), all of the networks showed similar levels of spatial overlap with the congruent HCP-networks. The exception was the Occipital Visual network which was more strongly correlated with its HCP-counterpart during visually related conditions (visual and visuomotor) than during rest (visual-rest difference score CI: -0.2173 to -0.0469, visuomotor-rest difference score CI: -0.2170 to -0.0464). Together these findings show that all ICNs, not only those activated by the experimental tasks [14], are spatially persistent across steady-states, despite the changes in brain activation.

### Local differences in connectivity profiles

Our previous analysis showed that the ICNs are broadly stable across different steady-state conditions, as identified using standard resting-state FC analysis approaches. Yet, as highlighted above, it is still likely that the task demands have a more localized impact on FC, which is insufficient to disrupt the ICNs global stability. To uncover more subtle differences across the connectivity profiles of task-relevant networks, we employed a dual regression analysis to interrogate connectivity changes in the networks most affected by the task activations.

**Fig 5:**
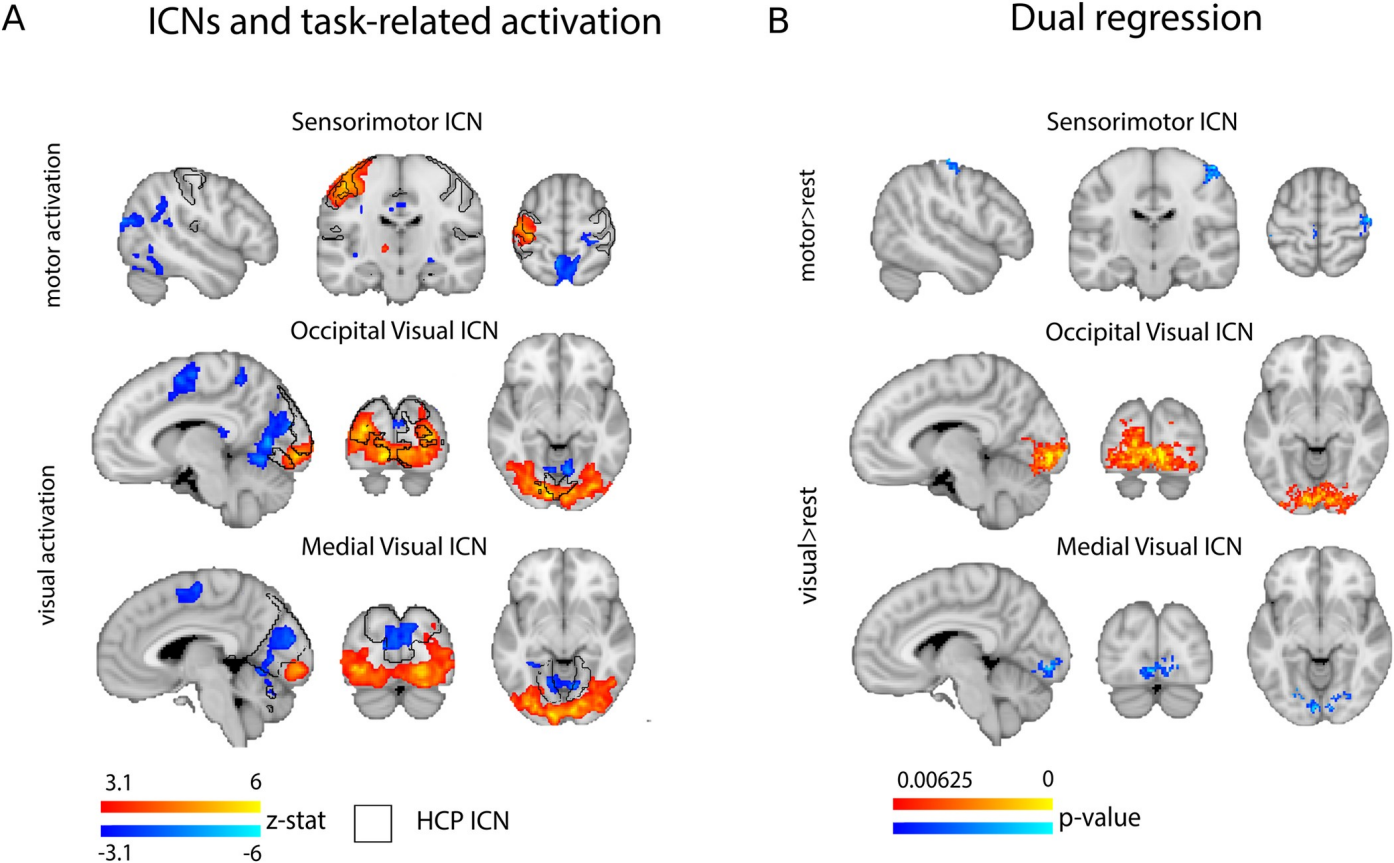
Intra-network FC differences between task and rest. (A) Brain regions activated by the motor condition overlap with the Sensorimotor ICN; brain regions activated during the visual condition overlap with the Occipital Visual ICN; and, brain regions deactivated during the visual condition overlap with the Medial Visual ICN. Brain (de)activation is shown in blue and red, the boundaries of the ICNs is illustrated by the black contour line. (B) Results of the dual regression analysis reveal: lower FC to the ipsilateral part of the Sensorimotor ICN during motor task; increased FC within the Occipital Visual ICN during visual task; and decreased connectivity between Medial Visual ICN and Occipital Visual ICN during the visual task.

We took advantage of our parametric design (visual activation on/off, motor activation on/off) to calculate one 2x2 ANOVA for each network. As no significant interactions were identified, we focused our analysis on the two main effects (see Methods). The Occipital Visual network overlapped with areas that were activated during the visual and visuomotor tasks. Accordingly, we found that the areas within the Occipital Visual network showed increase intra-network connectivity during those tasks, as compared to rest (Fig 5). In other words, the Occipital Visual network becomes more strongly connected to itself in the visual conditions, which may potentially underlie its stronger correspondence to its HCP-derived counterpart during visual and visuomotor steady-states (Fig 4C). The Medial Visual network overlapped with brain areas that were deactivated during the visual task. Accordingly, areas of this network showed reduced connectivity to the Occipital Visual ICN, which was activated during the visual conditions (Fig 5).

Finally, our motor task required the movement of only one hand, resulting in unilateral activation in the sensorimotor hand area contralateral to the task-related hand. Though the bilateral hand areas are typically coupled during resting state [11] the inactive hand (ipsilateral) area showed decreased connectivity with the sensorimotor network during the motor conditions. No other significant results were found for other contrasts and networks under the adjusted threshold (alpha<0.00625). Note that similar results were also found under higher attentional load in the visuomotor condition (see S1 Supporting Information), with additional FC modulations present in the Executive network. As such, areas that are activated/deactivated during the task show increases/decreases in network coupling during task compared to rest, although these changes are contained within the relevant intrinsic connectivity networks and may thus be attributed to changes in the amplitude of variance of the driving signal [35, 56].

## Discussion

In the present study, we investigated the effects of regional brain activation on FC, by comparing resting-state to steady-state task FC measurements. We used fMRI data from four steady-state tasks (Fig 1) to investigate modulations of FC due to changing task demands. We benefitted from using naturalistic visual and motor tasks that result in robust and consistent activation in sensorimotor cortex. For example, a similar motor task has been recently shown to produce the largest effect sizes for changes in BOLD activation, as compared to cognitive tasks [57].

In our first analysis, we looked at whole-brain changes in connectivity strength based on subject-specific activation profiles across nodes (Fig 3). Despite the fact that the activation was increased in a synchronized manner across task-specific nodes, we found no large-scale relation between FC and activation, suggesting that on average, FC changes may be better explained by network affiliation. To look at changes in the spatial attributes of FC we employed a data-driven approach based on ICA decomposition. We found that the resulting ICNs remain mostly undiminished during motor, visual, and visuomotor task conditions (Fig 4). This was also the case when we examined an additional task, designed to increase attentional load and to better integrate across the visual and motor conditions (Fig P in S1 Supporting Information). This analysis demonstrates that the ICNs, as identified using standard FC analysis practices, are not specific to resting state only but rather reflect the general state of functional brain organization. This observation is in accordance with previous studies postulating continuous intrinsic activity [2, 14, 58] and provides additional support for the idea that ICNs remain coupled within themselves even during task conditions [4, 31, 59]. Furthermore, we show that even those networks that are partially (or not at all) activated by the task largely maintain their functional integrity during the steady-states. Together, our findings suggest that gross functional brain structure is defined by a set of stable intrinsic networks that are present across both low-level tasks and rest.

A number of previous studies have shown that FC of ICNs can still be measured during both sensorimotor and cognitive tasks in addition to rest [14, 19, 29, 59–61]. For instance, Sun et al. seeded certain areas of the motor cortex during a bimanual motor task and found that they could reproduce a FC pattern similar to the sensorimotor ICN seen during rest [61]. Similarly, in macaques, Moeller et al. showed that independent component analyses of fMRI data acquired during movie-watching, rest and various visual tasks revealed FC networks that were highly similar across conditions [19]. However, note that in these examples, the task-activation pattern corresponded with the spatial properties of the related ICNs. In our experimental design, we varied the extent of activation within and across ICNs: the visual task, comprised of colorful and slowly moving shapes, was designed to activate the entire Occipital Visual network; the motor task, comprised of unilateral hand movements, was designed to only activate parts of the bilateral sensorimotor ICN; the combined visual and motor condition was designed to evoke a summation of visual and motor activations (an effect previously observed by [60]), providing opportunity to observe inter-network interactions. Despite this diversified experimental design, we found that the ICNs were largely invariant to changed activation (see below for a discussion of induced task-changes, as identified in our final analysis). The exception to this rule was the Occipital Visual ICN, where spatial changes in the connectivity pattern were identified in the visual task conditions, compared to non-visual conditions (Fig 4C). Our findings therefore demonstrate that the ICNs are robust to change, at least due to low task demands. This observation is consistent with recent evidence from cognitive tasks, which have been shown to introduce little variability to the gross structure of the FC networks [62]. Note however that other studies should determine whether these results can be replicated in a range of other steady-state tasks and paradigms, e.g. while activating only a proportion of the visual network and in tasks involving higher cognitive loads and/or fine motor precision.

While the node and ICA analyses described above were not sufficiently sensitive to identify task-related changes in intrinsic connectivity, more careful analysis (dual regression) did point at the task-variate changes. One of the most prominent explanations of the immutability of the ICNs across task conditions is that the resting state fluctuations are stable and linearly superimposed on the task activation, as first postulated by e.g. Arieli et al. [63] and Fox et al. [18]. More recent studies [17, 24, 62, 64, 65] suggested that the functional brain architecture during both rest and task performance is dominated by the ICNs that are superimposed on any potential task-evoked FC changes. In other words, task-evoked FC changes occur in the presence of an intrinsic functional network architecture that extends across many or all brain states. Thus, the activation-driven changes can only be resolved after removing the intrinsic connectivity components from the data [24, 64, 65] (though see also [66]).

Indeed, as stated above, using the dual regression procedure we did find significant differences in connectivity profiles arising from different steady-state tasks. In summary, we found that some brain areas activated by the task (i.e. within the Occipital Visual ICN during visual task) tended to become more connected to the network. Areas that become deactivated by a task (Medial Visual ICN in visual, viusomotor and attention tasks; ipsilateral hand area in motor, visuomotor and attention tasks) become decoupled from the activated network. The observed decrease in FC within the motor system was previously reported by Shah et al. [67] and Morgan and Price [68]. These authors hypothesized that this decrease in FC can be caused by the increased noise in the signal, induced by the finger tapping task. However, as those FC changes involve mainly the ipsilateral hemisphere (which does not activate during the task) rather than the entire sensorimotor network, we believe that the observed FC decrease originates from the lateralized activation characteristic of finger tapping (though we note that under higher attentional load the suppression was more extensive, see Fig P in S1 Supporting Information). All reported FC differences were, however, relatively localized to the activated/deactivated networks, suggesting that activation changes due to task demands only affect local connectivity within the network (as shown in Fig 5). This is consistent with the recent findings of Chauvin et. al [24] reporting higher level of significant within-network than between-network FC modulations due to cognitive involvement in the task. Exploring the linearity of the addition of the visual task to the motor task, we saw that there was relatively little interaction between the two conditions with respect to changes in connectivity, despite the fact that activation has been induced across both networks (see Fig R in S1 Supporting Information for inter-network changes in the attention task). These findings resonate with our previous conclusion, that network affiliation may be the most important aspect of functional connectivity. Indeed, a likely framework for explaining the differences in the ICN’s task-specific connectivity profile may be the changes in the amplitude of the driving signal [35]. Regardless, although relatively small, those connectivity changes are functionally meaningful and can potentially be used to distinguish between different cognitive tasks [29] and participants [15].

Despite widely established correspondence in rest-task network topography, recent studies have emphasized differences in FC patterns evoked by resting and task states [26, 28, 64, 69]. A common characteristic of most of the studies looking at activation-based changes in FC is that they use a block design and base their analyses on Psychophysiological Interactions (PPI). This procedure is based on the assumption that the global FC patterns can be initiated and stabilized within an order of seconds. However, it has been demonstrated that the characteristics of the spontaneous fluctuations change with time [70, 71] (see also [72–74] for considerations of the FC frequency band and [71] for dynamic functional connectivity). Here, we offer a paradigm that can help to ameliorate those confounds by using steady-state designs in which cognitive state is expected to be constant over time. Steady-state scans have been shown to be less susceptible to confounding factors than the block designs [31] and to produce more consistent FC results [3] In the current study, we thus employed a set of simple yet extensively studied motor and visual tasks, allowing us to examine the interaction between BOLD responses to a particular stimulus and FC changes. By varying the visual stimulus and moving fingers across each scan, our steady-state tasks were specifically designed to minimize effects of fMRI adaptation (also known as repetition suppression) which regardless, usually contributes to only a very small proportion of the BOLD signal [75]. Although performed continuously over a period of 5 minutes, we have found the steady-state tasks to be a good approximation of the task-localizer, in terms of reliably activating the same brain regions. Overall, we confirm previous observations that the intrinsic network architecture appears to be a canonical (default) state of the human brain’s functional network [21], with task demands having a small (yet potentially important) effect on this state when considered in terms of overall brain organization.

Our results provide two opportunities for methodological impact. First, we show that node-to-node correlations are insensitive to localized task-based changes in FC. Those changes were only significantly observed when utilizing a dual regression approach that effectively regresses out any contributions to the FC time-course that are shared by the other networks and conditions. This suggests that dual regression can be effectively used for unmasking local FC changes, providing alternative means to previously used approaches (e.g. inter-subject functional correlations [64, 76] or task potency [24]). Second, our findings suggest that steady-state designs can be used to study ICNs. Many studies have shown that motion is a major source of variability in FC studies that can lead to erroneous results when comparing groups of participants [77–79]. Moreover, it has been reported that task-based scans are associated with less head motion than classical resting-state scans [80, 81]. Despite these methodological benefits, task-based scans have been avoided in FC studies due to the assumption that intrinsic connectivity cannot be robustly measured under task conditions. Our data challenge this assumption: we show that the intrinsic connectivity structure dominates over task-evoked FC and is thus reliably present across multiple types of task-based brain states. Our findings therefore demonstrate that the gross features of intrinsic functional network structure can be reliably assessed, and compared between different populations, during various steady-states; and potentially even when different participants are engaged in different minimally demanding steady-state tasks. As suggested by Vanderwal et al. [80] this finding can facilitate data collection as it lowers the chances of participants falling asleep and significantly reduces their head movements. Furthermore, whereas resting state scans are largely uncontrolled (the final FC results can be altered by uncontrollable activations), steady-states paradigms offer a greater level of cognitive and experimental control, which may help to reduce variability in results or circumvent other confounds. However, we caution that this approach will drive localized changes, and as such researchers should be thoughtful when picking the most suitable task to engage their participants without affecting their networks of interest.

## Supporting information

S1 Supporting Information A single file containing all the supporting methods and results related to the attention task as well as all the supporting figures. (DOCX)

S1 Video The video of abstract shapes in motion, used during the visual and visuomotor steady-state tasks. (MP4)

S1 Data Raw (per-subject) results of the nodes analysis (related to Fig 3). All the values reported are mean correlation coefficients (calculated across time-courses of all pair-combination of nodes) computed separately for each subject, steady-state condition and number of activated nodes.
(XLSX)

S2 Data Raw results of the bootstrapping analysis (related to Fig 4). All the values reported in the file are mean spatial correlation coefficients (and associated standard deviations) calculated from 100 bootstrapped ICA decomposition, separately for each network of interest and each steady-state condition.
(XLSX)
