# Supplementary material for: Large-scale intrinsic connectivity is consistent across varying task demands
Source: PLoS One. 2019 Apr 10;14(4):e0213861. doi: 10.1371/journal.pone.0213861 (PMC6457563; doi:10.1371/journal.pone.0213861)
Supplement: S1 Supporting Information — (DOCX) [file pone.0213861.s001.docx]

**S1 Supporting Information**

**Supporting Methods**

## Stability of ICNs under higher attentional load

To explore the role of attentional load on the integrity of the intrinsic connectivity networks, all participants underwent an additional 5-minutes fMRI scan (attention task). During this scan participants repeated the combined visuomotor condition (see Methods), but were asked to change the finger-tapping direction (index-to-pinkie and reverse) whenever they noticed an irregularly appearing monochrome frame (on average, occurring every 30 seconds). This data was acquired, pre-processed and analyzed in the same way as detailed for the four main study conditions (see Methods), with the following exceptions: First, as we didn’t have a localizer task for this condition, we could not follow the node analysis. For the dual regression analysis we focused on an additional task-relevant network (Executive ICN), resulting in 5 networks of interest. Group statistical analysis was carried out using FSL randomize for each network of interest separately with four paired t-tests to assess differences between attention and rest (attention>rest and attention<rest), and attention and visuomotor conditions (attention>visuomotor and attention<visuomotor), with 5000 permutations and a threshold-free cluster enhancement. Due to exploratory nature of the analysis, uncorrected alpha values were used (alpha value = 0.05).

## Correlations across intrinsic connectivity networks

To further confirm the spatial correspondence of the steady-states ICNs to the HCP-derived resting state networks, we repeated our whole-brain correlations analysis (Fig 4B) after isolating the contribution of each of individual FC networks using dual regression. Dual regression was carried out in the same way as described in the Methods, however note that here we focused the analysis on all 9 identified ICNs. The resulting spatial maps of the 9 ICNs (separate for each participant and condition) were then correlated against the HCP-derived networks across the entire brain to obtain whole-brain correlation matrices. Individual participants’ matrices were then averaged for the visualization purposes (Fig S).

**Supporting Results**

## Stability of ICNs under higher attentional load

All of the canonical ICNs were found in the ICA decomposition of the attention task dataset. Similarly to the four main study conditions, spatial maps of the attention task networks showed high level of consistency with the HCP-derived resting state networks (see Fig N). Spatial correlations between congruent networks of the HCP and attention task were found to be considerably stronger than the correlations between incongruent networks (Fig O) (average r-value for the intra-network correlations: 0.416, average r-value for the inter-network correlations: 0.022). Note however, that the Sensorimotor and Executive ICNs were qualitatively more dispersed than in the other steady-state conditions, as evidenced by their higher spatial correlations with the incongruent HCP networks.

When compared to rest, all of the attention task-derived networks showed similar levels of spatial overlap with their corresponding HCP networks (see Fig O). The exception was the Occipital Visual Network, which as observed in the visual and visuomotor steady-states, correlated mote strongly with its HCP-counterpart during all visually related conditions (attention task-rest difference score CI: -0.2297 to -0.0665). Interestingly, we have also observed that during the attention task the Executive Network tended to decrease in its spatial correspondence to the HCP-counterpart. Although this result failed to reach significance, it suggests that some subtler local FC changes may be occurring within this network under higher attentional load.

To examine more subtle differences across the connectivity profiles of task-relevant networks, we employed a dual regression analysis. Note that due to the exploratory nature of this supplementary analysis we did not employ any corrections across the 20 comparisons and therefore any results should be interpreted with caution. Consistent with the other visual conditions, we found that during the attention task, Occipital Visual Network, activated during the task, showed stronger connectivity to itself, while Medial Visual Network, which overlaps with the deactivated cluster, decreased in its connectivity to itself and to the Occipital Visual Network. Similarly, both Sensorimotor and Executive Networks became less coupled with themselves during the attention task (see Fig P). Note however, that while in the other motor conditions, the decrease in the FC within the Sensorimotor network was observed unilaterally (see Results), here the observed effect was bilateral. These findings largely mirror the results reported for the other steady-state conditions (see Results) and support our hypothesis that the areas that activate/deactivate during task may show increase/decrease in network coupling during task compared to rest, with those changes largely contained within the relevant ICNs. However, since the study was not designed to determine the role of cognitive load on FC stability, this important aspect should be considered in future research more thoroughly.

Finally, to isolate the effects of the increased attentional load on the local FC changes, we have looked at the contrast between the attention task and the visuomotor task. We found further decrease in connectivity within the Medial Visual Network (as observed above) to be driven by the induced attentional load. Furthermore, higher attentional load resulted in the Default Mode Network becoming less connected to the Lateral Visual Network (activated during the task), as compared to the visuomotor task (see Fig P; see Table A for other small clusters found across the 20 contrast maps).

## Correlations across intrinsic connectivity networks

Isolating the contribution of each of the ICNs to the intrinsic FC pattern resulted in greater specificity in the spatial correspondence to HCP-derived networks (no inter-network correlations, see Fig R). Note however that here the average correlation strength between the steady-states network and the HCP-derived networks was lower in magnitude than in our original analysis (Fig 4B).

**Supporting Figures and Tables**

**Table A. Locations and sizes of small clusters of FC differences found between attention and visuomotor tasks, as revealed by dual regression analysis**

| **ICN** | **Contrast** | **MNI coordinates of cluster’s centroid** | **Cluster’s size** |
| --- | --- | --- | --- |
| **Occipital** | Attention<Visuomotor | x: 65.77, y: 28.00, z: 32.44 | 9 voxels |
| **Sensorimotor** | Attention<Visuomotor | x: 68.00, y: 85.50, z: 30.00 | 2 voxels |
| **Executive** | Attention<Visuomotor | x: 44.64, y: 26.86, z: 54.86 | 14 voxels |


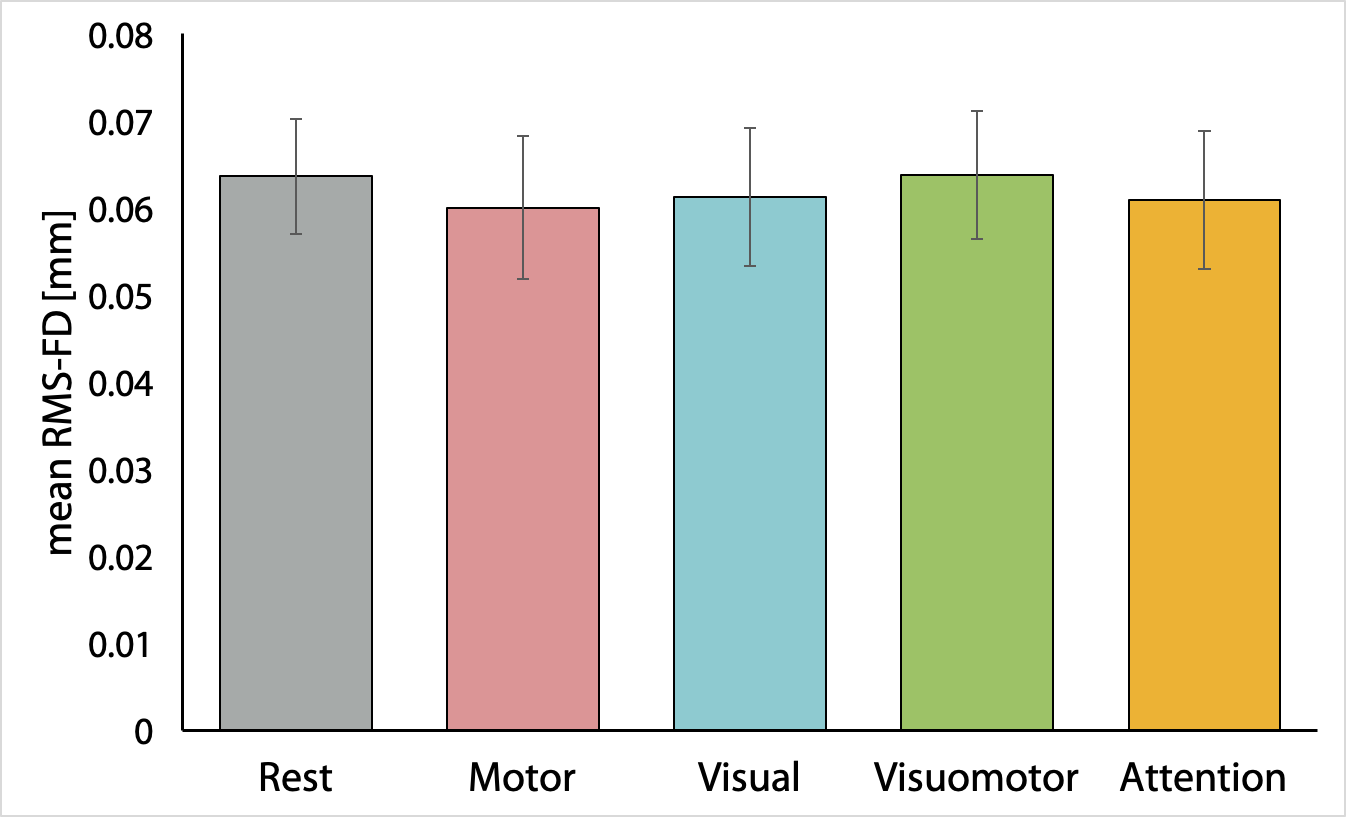


**Fig A. Average head movements during each of the steady-state scans**. Head motion was quantified using the root mean square of the frame-wise displacement computed by MCFLIRT. No statistically significant differences were found between the steady-state scans.

**Fig B. Individual participant’s activation maps overlaid on the HCP multi-modal parcellations.** As visualised here, individual activation clusters are often smaller in size and not perfectly aligned with the original HCP parcellations. This observation led us to further customize the HCP ROIs based on individual activation profiles (see Methods).


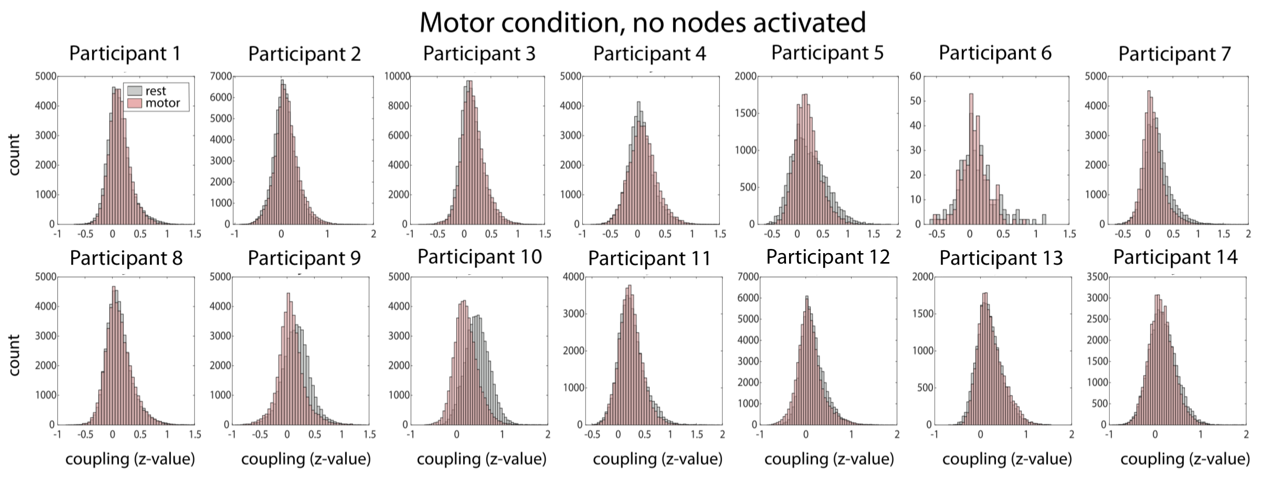


**Fig C.** Fisher z-transformed correlation coefficients between pairs of nodes, where none of the nodes is activated during the motor steady-state task, are displayed separately for each participant in the form of a histogram.


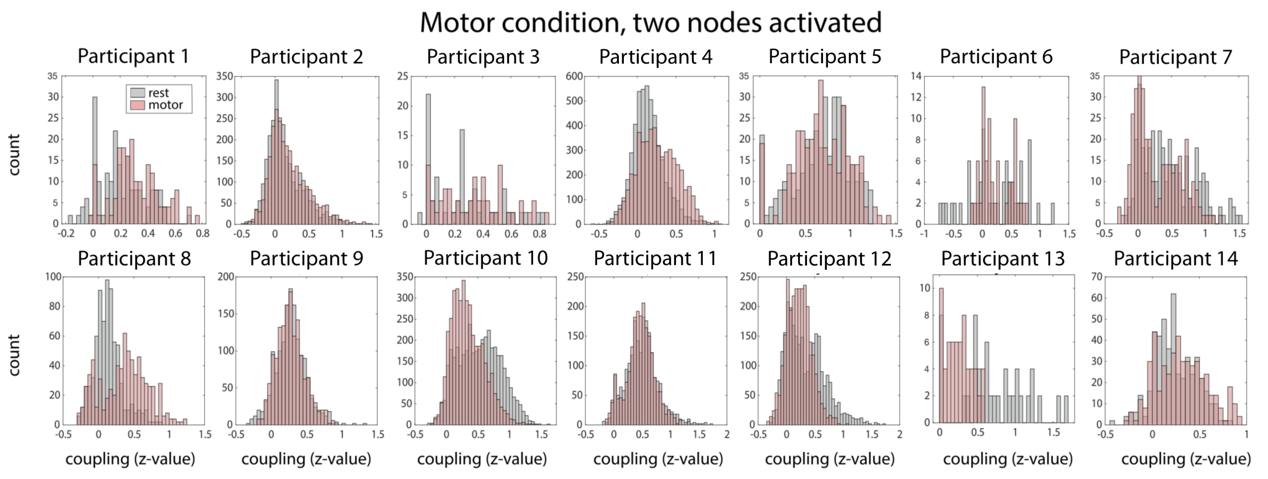


**Fig D.** Fisher z-transformed correlation coefficients between pairs of nodes, where both of the nodes are activated during the motor steady-state task, are displayed separately for each participant in the form of a histogram.

**
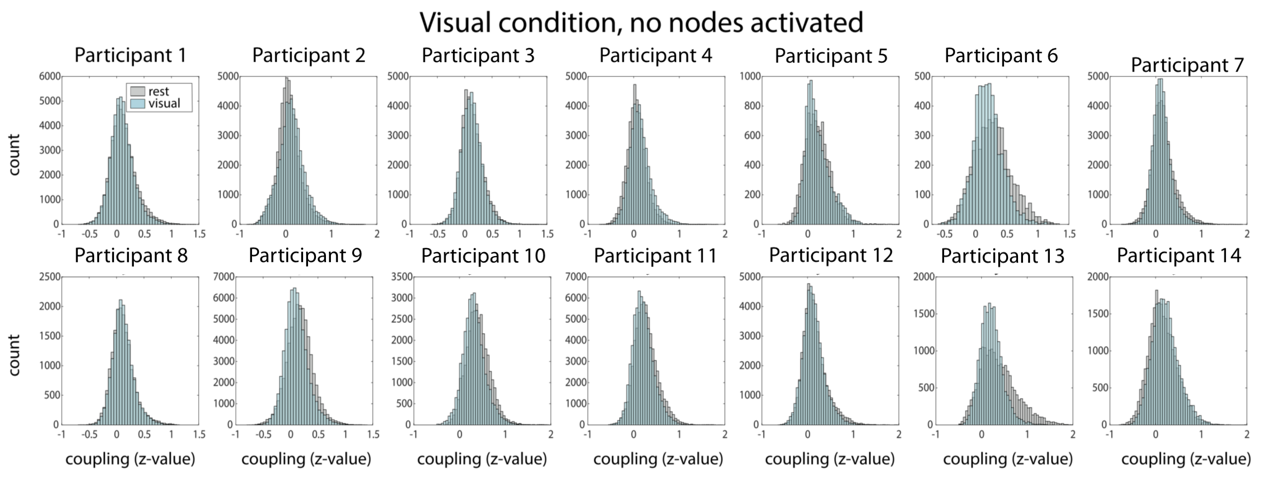
**

**Fig E.** Fisher z-transformed correlation coefficients between pairs of nodes, where none of the nodes is activated during the visual steady-state task, are displayed separately for each participant in the form of a histogram.


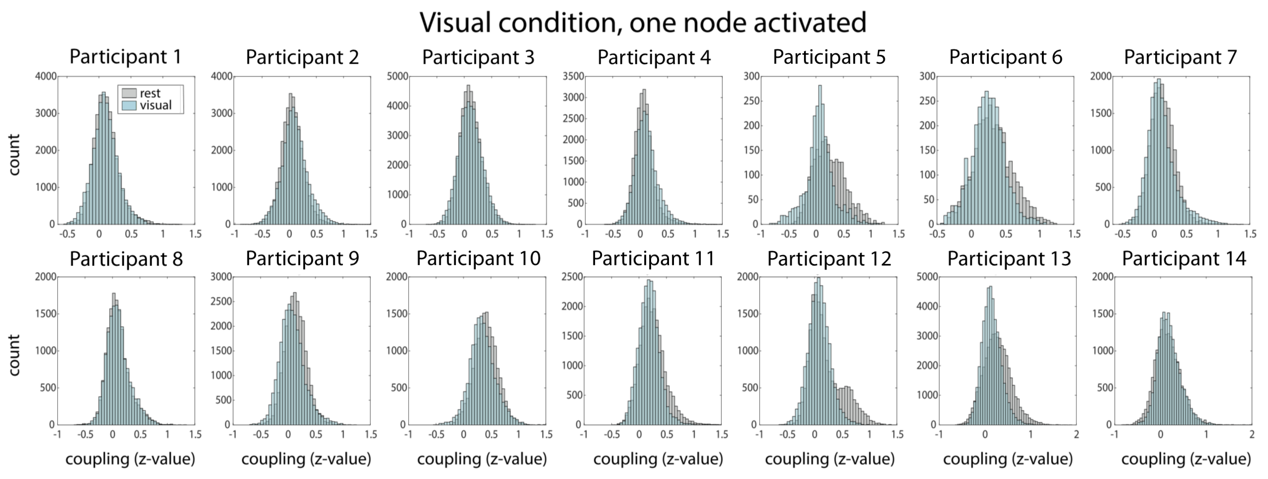


**Fig F.** Fisher z-transformed correlation coefficients between pairs of nodes, where only one of the nodes is activated during the visual steady-state task, are displayed separately for each participant in the form of a histogram.


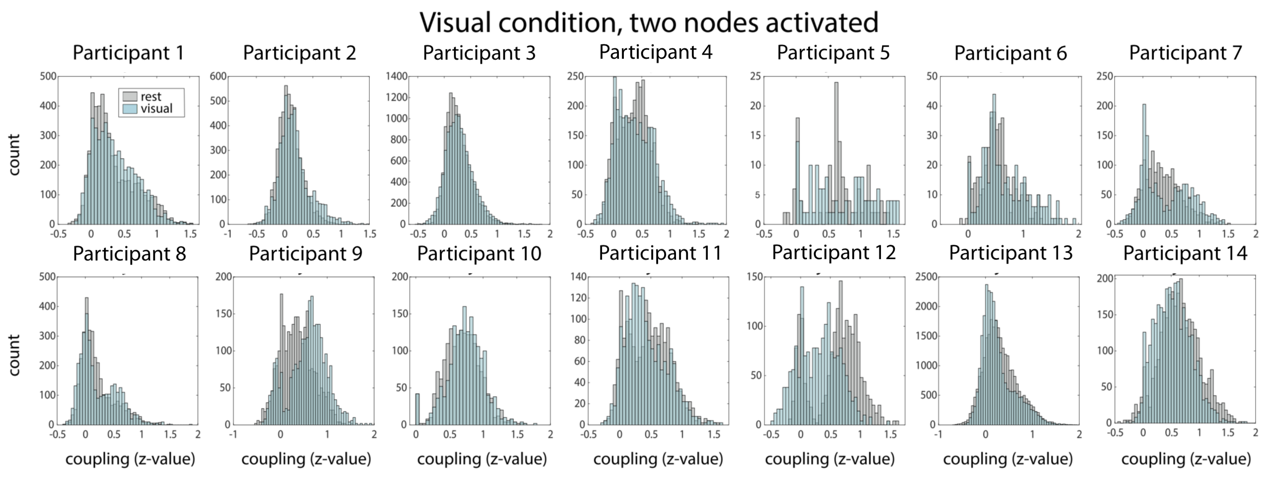


**Fig G.** Fisher z-transformed correlation coefficients between pairs of nodes, where both of the nodes are activated during the visual steady-state task, are displayed separately for each participant in the form of a histogram.


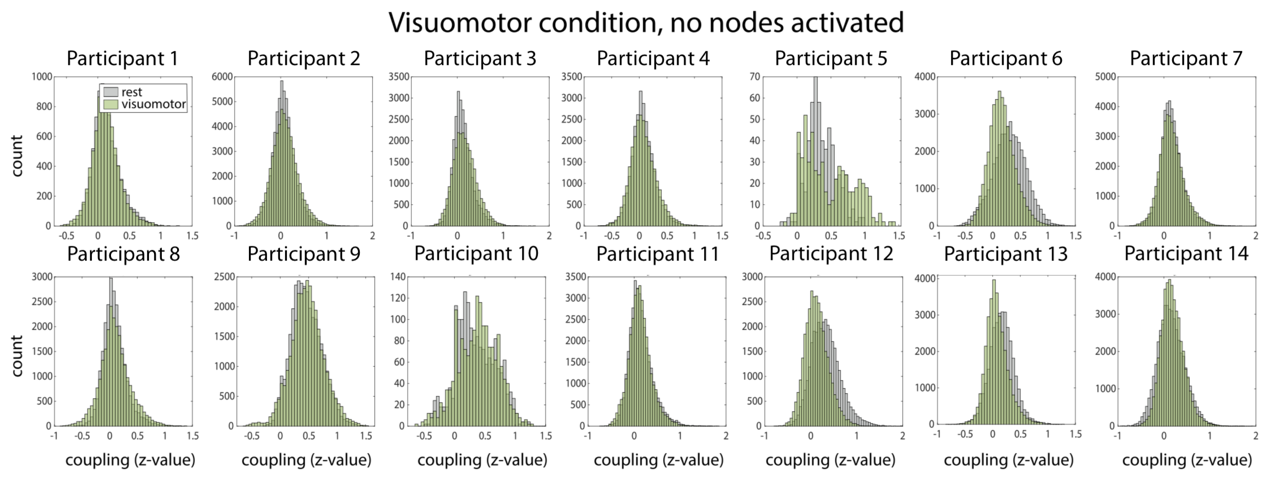


**Fig H.** Fisher z-transformed correlation coefficients between pairs of nodes, where none of the nodes is activated during the visuomotor steady-state task, are displayed separately for each participant in the form of a histogram.


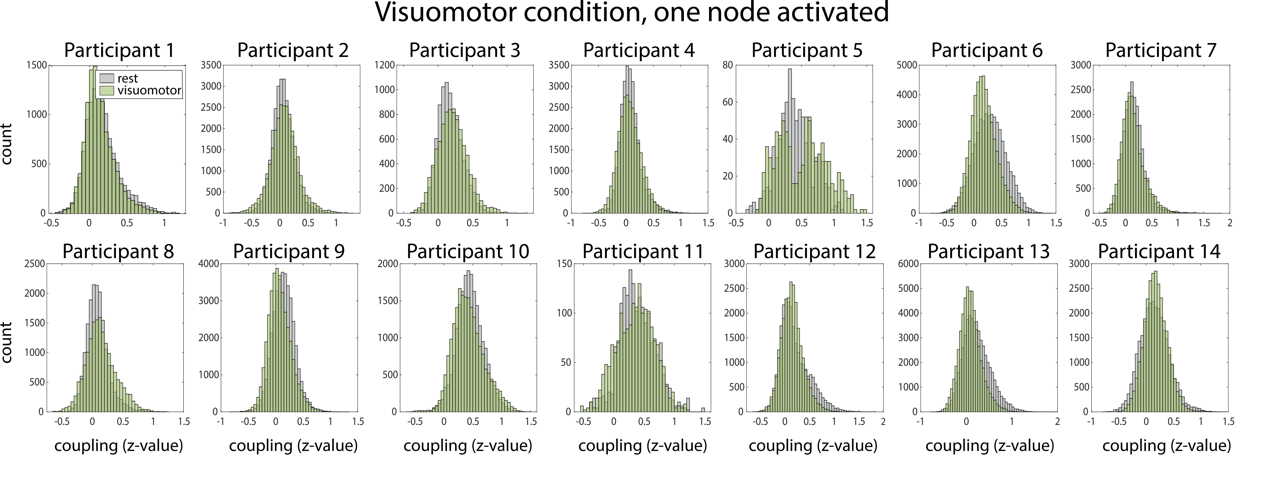


**Fig I.** Fisher z-transformed correlation coefficients between pairs of nodes, where only one node is activated during the visuomotor steady-state task, are displayed separately for each participant in the form of a histogram.

**
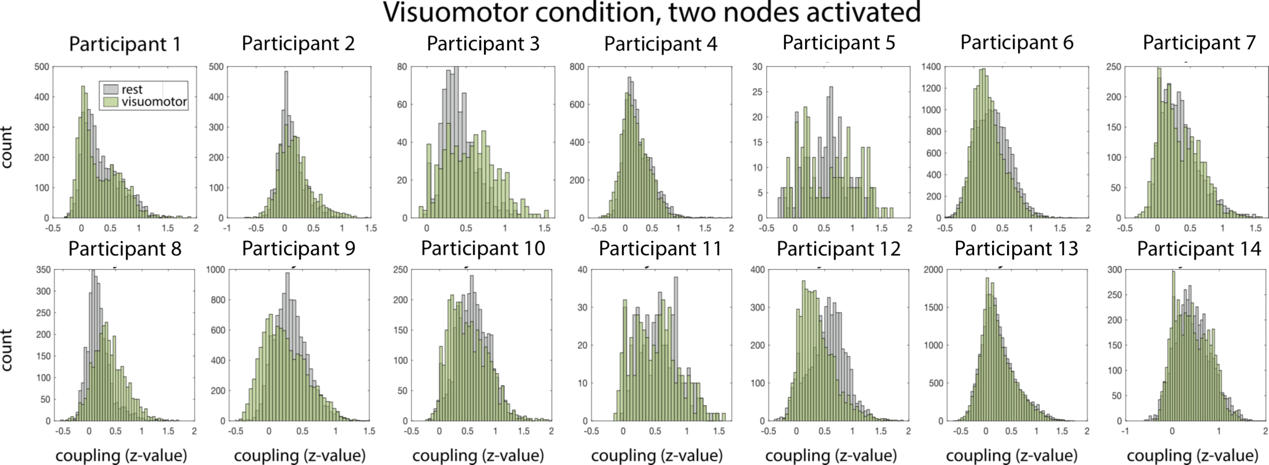
**

**Fig J.** Fisher z-transformed correlation coefficients between pairs of nodes, where both of the nodes are activated during the visuomotor steady-state task, are displayed separately for each participant in the form of a histogram.

**Fig K.** Spatial maps of all 9 major ICNs extracted from the resting state data (depicted in red-yellow scale) overlaid on the same ICNs extracted from the HCP data (depicted as black contours).

**Fig L.** Spatial maps of all 9 major ICNs extracted from the motor condition (depicted in red-yellow scale) overlaid on the same ICNs extracted from the HCP data (depicted as black contours).

**Fig M.** Spatial maps of all 9 major ICNs extracted from the visual condition (depicted in red-yellow scale) overlaid on the same ICNs extracted from the HCP data (depicted as black contours).

**Fig N.** Spatial maps of all 9 major ICNs extracted from the visuomotor condition (depicted in red-yellow scale) overlaid on the same ICNs extracted from the HCP data (depicted as black contours).


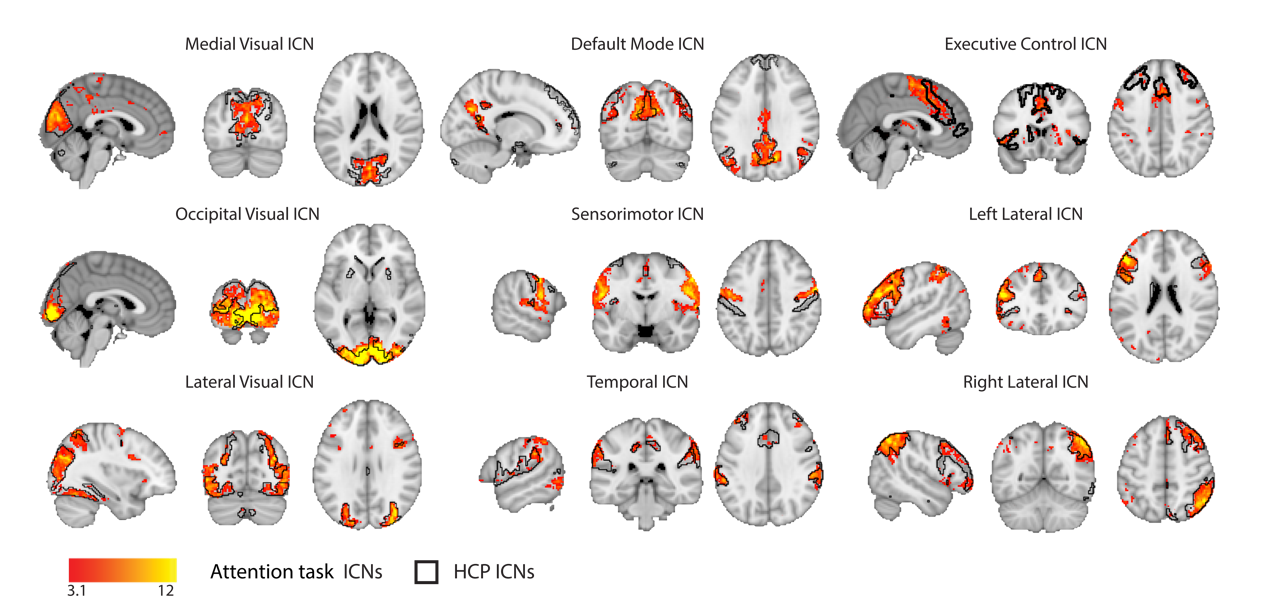


**Fig O.** Spatial maps of all 9 major ICNs extracted from the attention task condition (depicted in red-yellow scale) overlaid on the same ICNs extracted from the HCP data (depicted as black contours).


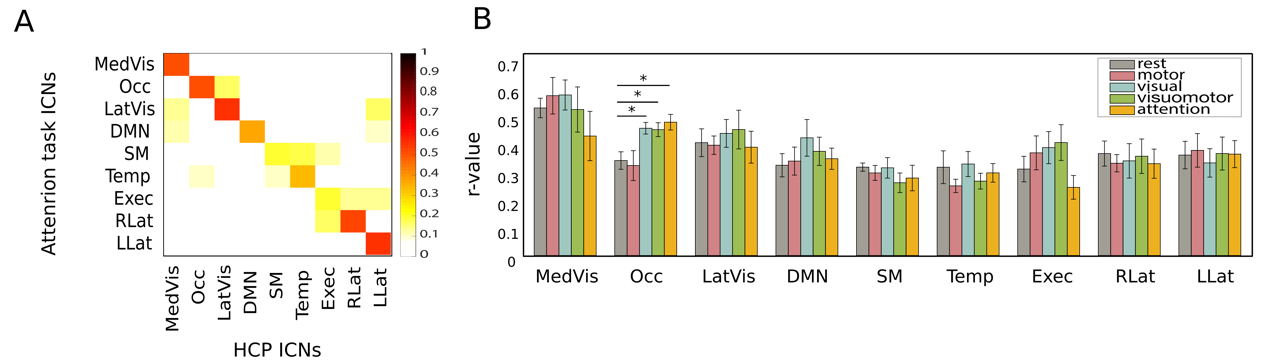


**Fig P. Spatial variability of ICNs in the attention task condition** (A) Whole brain correlation matrices of 9 major ICNs from the HCP data and their counterparts found in the attention task data. Each ICN is correlated with all other ICNs. (B) Bar graph depicting mean spatial correlation coefficients (calculated from 100 bootstrapped ICA decomposition) of 9 major ICNs extracted from each of the five steady-state conditions to their HCP counterparts. Note that only the Occipital Visual ICN (Occ) shows significant differences in its spatial correspondence to the HCP’s Occipital Network. Asterisks denote significance as determined using bootstrap percentile confidence intervals (see Methods). MedVis stands for Medial Visual ICN, Occ – Occipital Visual ICN, LatVis – Lateral Visual ICN, DMN – Default Mode Network, SM – Sensorimotor ICN, Temp – Temporal ICN, Exec – Executive ICN, RLat – Right Lateral ICN, LLat – Left Lateral ICN.


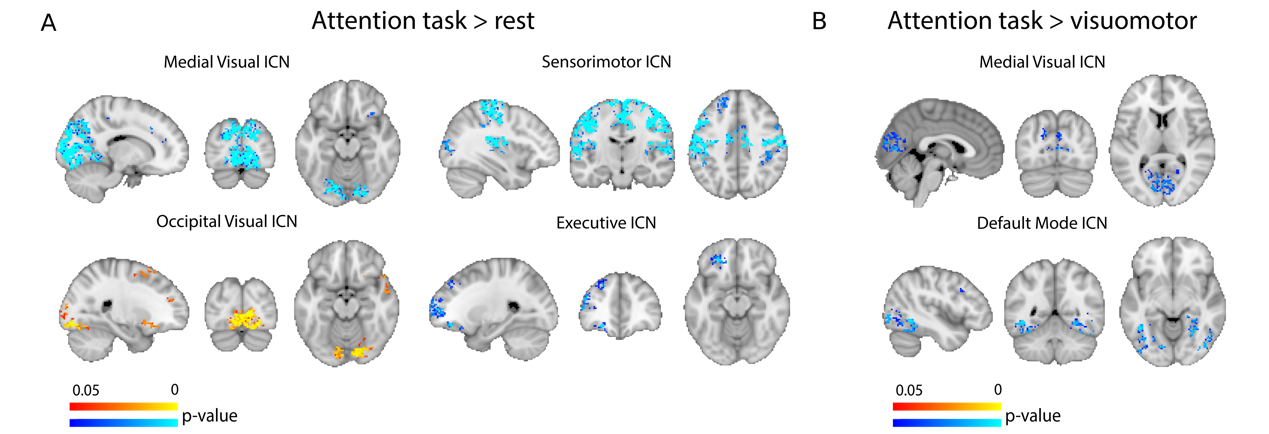


**Fig R. Intra-network FC differences induced during attention task** (A) Comparison between attention task and rest: results of the dual regression analysis reveal: decreased connectivity between Medial Visual ICN and Occipital Visual ICN; decreased FC within Medial Visual ICN; increased FC within the Occipital Visual ICN during visual task; decreased FC within the Sensorimotor ICN; and decreased FC within Executive ICN during the attention task. (B) Comparison between attention task and visuomotor task: results of the dual regression analysis reveal: lower FC within the Medial Visual ICN; and lower connectivity between Default Mode ICN and Lateral Visual ICN under higher attentional load.


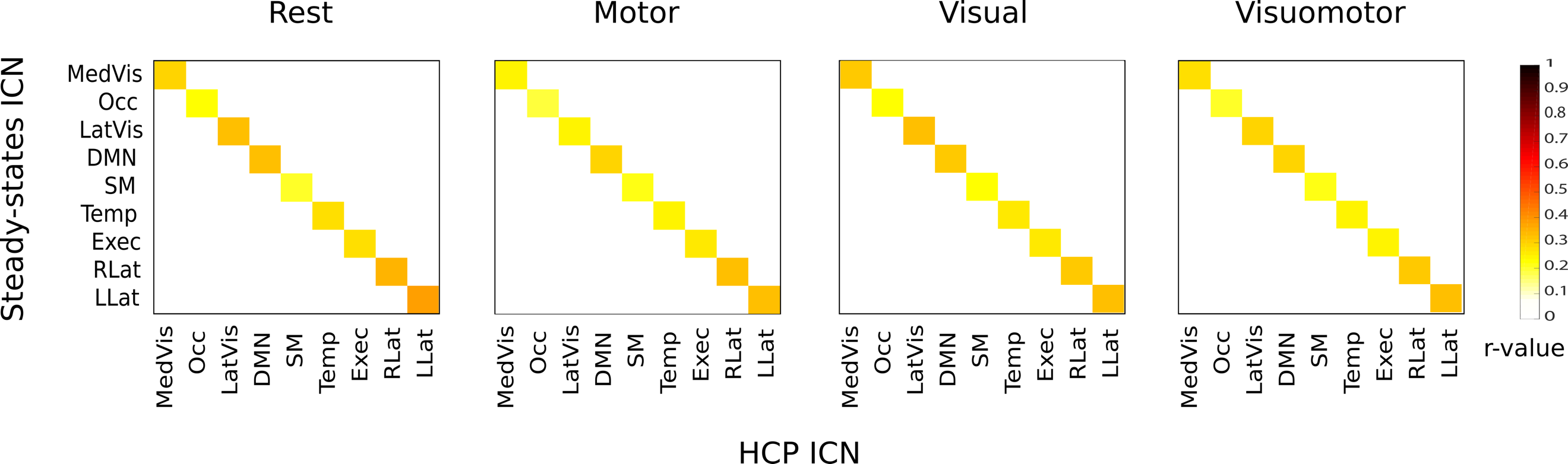


**Fig S. Spatial variability of ICNs after isolating the individual networks’ contribution to the intrinsic FC pattern.** Whole brain correlation matrices of 9 major ICNs from the HCP data and their counterparts found in the steady-states. Each ICN is correlated with all other ICNs.
